# Adherence to methadone maintenance treatment and associated factors among patients in Vietnamese mountainside areas

**DOI:** 10.1186/s13011-017-0115-4

**Published:** 2017-06-08

**Authors:** Long Hoang Nguyen, Huong Thu Thi Nguyen, Huong Lan Thi Nguyen, Bach Xuan Tran, Carl A. Latkin

**Affiliations:** 1 School of Medicine and Pharmacy, Vietnam National University, Hanoi, Vietnam; 2 Institute for Preventive Medicine and Public Health, Hanoi Medical University, Hanoi, Vietnam; 3 Thanh Nhan Hospital, Hanoi, Vietnam; 4 Institute for Global Health Innovations, Duy Tan University, Da Nang, Vietnam; 5 Johns Hopkins Bloomberg School of Public Health, Baltimore, MD USA

**Keywords:** Adherence, Methadone, MMT, Mountainous, Vietnam

## Abstract

**Background:**

Medication adherence is essential to achieve successful methadone maintenance treatment (MMT). However, treatment adherence among MMT patients in the mountainous setting in Vietnam has not been yet investigated. This study aimed to explore the medication adherence and associated factors in MMT patients in Tuyen Quang, a mountainous province.

**Methods:**

A cross-sectional survey was conducted in two MMT clinics namely Tuyen Quang and Son Duong. Convenience sampling method was used to recruit patients. Adherence to MMT was assessed by using three questions: 1) number of days that they missed doses in the last 4 days; 2) whether they missed doses during the last weekend and 3) when they missed a dose within the last 3 months. Adherence was considered optimal if patients reported ‘no’ to three questions. Socioeconomic status, health status (measured by EuroQol-5 Dimensions – 5 Levels – EQ5D5L and Visual analogue scale – VAS), substance use and abuse and methods to support adherence were also collected.

**Results:**

Among 241 patients, 34.4% reported optimal adherence. Self-help was the most popular (89.2%) method used to support adherence. Risk factors of missing doses and suboptimal adherence included higher education and economic status; being a worker/farmer; longer duration of treatment; and suffering pain/discomfort and anxiety/depression. Protective factors were older age, having problems in usual activities/self-care, higher EQ-VAS and EQ-5D index; and reminded by mobile phone and family members.

**Conclusions:**

This study found a high sub-optimal adherence rate among MMT patients in a mountainous setting in Vietnam. Measuring adherence by using several simple items could be used periodically to monitor the treatment adherence in the clinical setting. Family and mobile phone support would have a potential role in supporting patients to adhere treatment.

## Background

Ensuring the sustainability of a health program needs great efforts to maintain the program’s benefits regardless of funding received [1]. As methadone maintenance treatment (MMT) has been an integral part of HIV elimination plan worldwide [2, 3], efforts to optimize the effectiveness of this intervention have been growing in the recent years, including strategies to improve the retention and adherence of MMT patients. Methadone is a *μ*-opioid receptor agonist that has been shown helpful in reducing illicit drug use, drug-related risk behaviors and crimes, as well as promoting health and well-being among opioid dependence persons [4, 5]. However, because MMT is a slow-onset and long-acting medication, non-treatment adherence may lead to the increased risk of suffering withdrawal symptoms, drug relapse and overdose due to loss of tolerance [6, 7]. Therefore, along with receiving sufficient doses, long-term adherence is strictly required for MMT patients to maximize the effectiveness of intervention [8, 9].

Nonetheless, non-adherence with methadone treatment is frequently noted in global reports. The lowest proportion of non-adherence is observed in America countries with only 17% in the United States and 15.5% in Canada [10, 11]. Conversely, this rate is much higher in other regions. In Europe, surveys in France and the United Kingdom showed that 65.2% and 42% patients did not adhere the treatment, respectively [8, 12]. Meanwhile, in Asia, studies in China suggests that the proportion of MMT patients having poor adherence were from 36.3% to 88.2% [13, 14]. One longitudinal study in 2009 in Vietnam indicated that the rate of missing dose for 1–2 days increased from 18.4% in the first 3 months to 41.4% after 24 months [15]. Determinants of non-adherence varied across the settings. Overall, low socioeconomic status, polysubstance abuse (e.g. alcohol and drug use), deficient methadone dose, long distance to MMT clinics and being dissatisfied with MMT services are found to be associated with non-adherence among MMT patients [8, 12-14, 16]. Thus, to increase the effectiveness of MMT program, it is necessary to measure treatment adherence as well as associated factors, which may help policy makers and program managers to provide timely and tailored solutions for each patient.

The Vietnamese Government has given a strong commitment to providing MMT service to 80,000 opioid dependence persons in the coming years [17]. To date, 46,000 drug users have been offered MMT at 251 clinics nationwide [18]. However, the maintenance of MMT program has been confronting a great challenge when the Vietnam Government can only subsidize 50% of resource needed in the coming years due to the rapid cut of international donors [17]. In this context of limited resources, data on adherence pattern can be used to optimize the advantages of MMT program, as well as control the quality of MMT service and serve as an indicator for the resource allocation.

However, little attention is paid to explore the medication adherence and its related factors, especially in mountainous settings. Previous work conducted in two Vietnamese metropolitans suggests the increasing non-adherence overtime, but this study did not identify the determinants of non-adherence [15]. Therefore, this study aimed to explore adherence among MMT patients. The findings of this study may enhance our understanding of adherence to MMT as well as suggest methods to improve the effectiveness of MMT program.

## Methods

### Study setting and sampling method

From May to August 2016, we conducted a cross-sectional study in Tuyen Quang, an epicenter of Northern Vietnam with approximately 1100 registered drug users and 388 patients enrolling MMT program. There are three MMT clinics available in this province, including Tuyen Quang, Son Duong, and Yen Son. Because Yen Son clinic had only nine patients, we involved two remaining clinics: Tuyen Quang as being representative of the urban area and Son Duong as being representative of the rural area. The characteristics of MMT service in each clinic were described in Table 1.

**Table 1:** Characteristics of MMT service in each selected clinic

Clinic	No. staffs	Opening hour	No. patients	Setting
Tuyen Quang city	11	7:00 a.m. to 17:00 p.m. (Weekdays) 7:00 a.m. to 11:30 a.m. (Weekend)	284	Urban area
Son Duong	9	7:00 a.m. to 17:00 p.m. (Weekdays) 7:00 a.m. to 11:30 a.m. (Weekend)	95	Rural area

Study participants were patients who 1) were receiving methadone medication at selected sites, 2) were available at the clinics during the study period, 3) were 18 years of age or above, 4) had the ability to complete a questionnaire, 5) agreed to participate, and 6) were able to provide informed consent. Convenience sampling methods were used to recruit patients. A total of 241 patients agreed to be in the study. Because there were only five female patients in all three clinics, the participants in our study were only male.

### Measurements and Instruments

A patient who met eligible criteria would be invited to a designated room to ensure the privacy, then asked to enroll in the study and sign the written informed consent form. Face-to-face interviews were conducted by masters of public health students and staff from Hanoi Medical University using a structured questionnaire. Each interview was prolonged within 20–25 min. The patients did not receive any incentives or travel reimbursements for participating in this study. There were no clinic staffs participating in the study as interviewers to avoid any potential bias.

The main outcomes of this study were treatment adherence. First, patients reported their adherence in the last 7 days by using a 100-point visual analog scale (VAS), from 0 “complete non-adherence” to 100 “perfect adherence.” A threshold 90% was used to detect the optimal adherence [13, 19]. Then, we asked participants to report their adherence according to the standard of Ministry of Health (MoH) by using three questions: 1) number of days that they missed doses in the last 4 days; 2) whether they missed doses in the last weekend and 3) when they missed the last dose within the last 3 months. Adherence was considered optimal if patients reported ‘no’ to three questions, and suboptimal if they answered ‘yes’ or “don’t remember” to any question [9]. Finally, we collected information regarding methods to support adherence in MMT patients.

To identify the determinants of adherence, we included some covariates namely socio-economic status (age, education level, marital status, employment status, ethnicity and monthly household income), health status and substance abuse (alcohol use, tobacco smoking, and concurrent illicit drug use).

To measure HRQOL, we employed a well-validated tool namely EuroQol - five dimensions - five levels (EQ-5D-5 L), which assessed five components: Mobility, Self-care, Usual activities, Pain/Discomfort and Anxiety/Depression. Each domain had five response levels from no problems to extreme problems, producing a total of 3125 health indexes. A Thailand interim scoring for EQ-5D-5 L was used to calculate these indexes because there was no value set for Vietnamese population’s preference [20, 21]. We also utilized a 100-point visual analogue scale (EQ-VAS) to measure self-reported HRQOL, ranging from 0 “The worst health state that you can imagine” to 100 “The best health state that you can imagine.” Furthermore, we also asked patients to report their HIV serostatus and whether they received antiretroviral therapy (ART).

Regarding alcohol use, we used the Alcohol Use Disorders Identification Test-Consumption (AUDIT-C) to assess the level of alcohol abuse [22]. This tool contained three questions with the sum of score ranging from 0 to 12. Participants were classified as hazardous drinkers if they had score ≥ 4 for male and ≥3 for female [22]. In terms of cigarette smoking, we asked the patients to report whether they ever smoked at least 100 cigarettes in entire life and currently smoked in the last 30 days. The patients were classified as a current cigarette smoker if they had these features. We also collected self-reported information about the duration of MMT, whether they currently used illicit drugs and their history of drug rehabilitation.

### Statistical analysis

STATA software version 12.0 (Stata Corp. LP, College Station, United States of America) was used to analyze data. We ignored the missing data because of the fact that the highest percentage of missing data was in the VAS variable for adherence (with 12 people not reporting = 5.0%). Schafer and Bannett indicated that the missing rate of 5% or less did not influence the results [23, 24]. Therefore, our missing rate is acceptable.

T-test (for data with normal distribution), Chi-square test and Fisher’s exact test were used to detect the differences of variables between two durations of treatment: in the first 12 months (short term) and more than 12 months (long term). Our main hypothesis is that patients undergoing long-term treatment have lower medication adherence compared to their counterparts. A *p*-value <0.05 (two-tails) was used to identify statistical significance. In this study, a stepwise backward selection strategy was applied along with multivariate Logistic and Tobit regressions to have reduced models. This strategy used threshold with the log-likelihood ratio test to have predictors with *p*-values of <0.2 included.

## Results

Among 241 patients, most of the respondents were 35 to 50 years old (62.7%), had higher education or above (53.0%), lived with spouse/partner (62.3%) and were employed (93.6%). About three-quarters (75.7%) of the patients were smokers, while 18.3% and 13.4% were hazardous drinkers and concurrent drug users, respectively. Regarding health status, less than 15% of patients had problems in mobility, self-care, and usual activities, respectively, while 19.9% had pain/discomfort and 25.9% had anxiety/depression. About one-fourth of patients reported being HIV positive and currently received ART treatment (Table 2).

**Table 2:** Characteristics of respondents

Characteristics	MMT duration ≤12 months		MMT duration >12 months		Total	
	*n*	%	*n*	%	*n*	%
Total	102	42.3	139	57.7	241	100.0
Age group						
• Under 35	29	28.4	26	18.7	55	22.8
• 35 to 50	60	58.8	91	65.5	151	62.7
• Above 50	13	12.8	22	15.8	35	14.5
Education						
• < High school	50	49.0	51	35.5	111	47.1
• High school	48	47.1	58	43.3	106	44.9
• > High school	4	3/9	15	11.2	19	8.1
Marry status						
• Single	22	21.6	32	23.9	54	22.9
• Living with spouse/partner	61	59.8	86	64.2	147	62.3
• Divorced/Separate/Widow	19	18.7	16	12.0	35	14.8
Employment						
• Unemployed	9	8.8	6	4.5	15	6.4
• Self-employed	45	44.1	67	50.0	112	47.5
• Workers/Farmers	19	18.6	19	14.2	38	16.1
• Others	29	28.4	42	31.3	71	30.1
Location						
• Tuyen Quang	65	63.7	102	73.4	167	69.3
• Son Duong	37	36.3	37	26.6	74	30.7
Smoking status	72	72.7	102	77.9	174	75.7
Hazardous drinking	12	11.8	32	23.0	44	18.3
Current drug use	20	20.0	11	8.3	31	13.4
Number of drug rehabilitation						
• 0 times	18	17.7	17	12.2	35	14.5
• 1 times	29	28.4	43	30.9	72	29.9
• 2 times	19	18.6	32	23.0	51	21.2
• > 2 times	36	35.3	47	33.8	83	34.4
Having problems in mobility	14	13.7	19	14.2	33	14.0
Having problems in self-care	12	11.8	13	9.7	25	10.6
Having problems in usual activities	15	14.7	19	14.2	34	14.4
Pain/Discomfort	16	15.7	31	23.1	47	19.9
Anxiety/Depression	26	25.5	35	26.1	61	25.9
HIV positive	24	24.2	35	26.5	59	25.5
Currently receiving ART	23	22.8	30	22.7	53	22.8
	Mean	SD	Mean	SD	Mean	SD
EQ5D5L index	0.9	0.20	0.9	0.21	0.9	0.20
EQ VAS	81.9	16.3	81.8	14.5	81.8	15.3

Table 3 illustrates that 89.2% patients preferred self-help groups as an approach to support adherence. It was also chosen as the most effective way to support taking drugs with 67.7%. Additionally, most of the respondents would prefer wife/husband/lover to support them to adhere to medication (53.0%).

**Table 3:** Method to support adherence

Characteristics	MMT duration ≤12 months		MMT duration >12 months		Total		*p*-value
	*n*	%	*n*	%	*n*	%	
Methods to support adherence							
• Set the alarm clock^a^	14	13.7	19	13.7	33	13.7	0.99^*¶*^
• Set the mobile phone alarm^b^	18	17.7	26	18.7	44	18.3	0.83^*¶*^
• SMS reminder^c^	7	6.9	10	7.2	17	7.1	0.92^*¶*^
• Reminded by relatives^d^	33	32.4	35	25.2	68	28.2	0.22^*¶*^
• Reminded by health workers^e^	0	0.0	2	1.4	2	0.8	0.33^†^
• Self-help^f^	94	92.2	121	87.1	215	89.2	0.21^*¶*^
• Select appropriate work-shifts^g^	1	1.0	4	2.9	5	2.1	0.30^†^
• None^h^	1	1.0	5	3.6	6	2.5	0.31^†^
Most effective manner to not miss doses^i^							
• Set the alarm clock	3	3.1	11	8.2	14	6.0	0.16^†^
• Set the mobile phone alarm	8	8.2	6	4.5	14	6.0	
• SMS reminder	1	1.0	4	3.0	5	2.2	
• Reminded by relatives	21	21.4	18	13.4	39	16.8	
• Self-help groups	65	66.3	92	68.7	157	67.7	
• Select appropriate work-shifts	0	0.0	1	0.8	1	0.4	
• None	0	0.0	2	1.5	2	0.9	
Invite people to support adhering MMT^k^							
• Wife/Husband /Lover	52	53.1	71	53.0	123	53.0	0.45^*¶*^
• Parents	18	18.4	16	11.9	34	14.7	
• Others	6	6.0	10	7.4	16	7.2	
• Do not want to invite	4	4.1	12	9.0	16	6.9	
• No one engaged	18	18.4	25	18.7	43	18.5	

^*¶*^ *χ* ^2^ *;* ^†^ *fisher’s exact test;*

^a^Pearson chi-squared test = 0.0002; ^b^Pearson chi-squared test = 0.0441; ^c^Pearson chi-squared test = 0.0099; ^d^Pearson chi-squared test = 1.4945; ^e^Fisher’s exact test = 0.510; ^f^Pearson chi-squared test = .15939; ^g^Fisher’s exact test = 0.399; ^h^Fisher’s exact test = 0.406; ^i^Fisher’s exact test = 0.157; ^k^Pearson chi-squared test = 3.6949

Table 4 indicates that 9.1% patients missed taking methadone in the last 4 days and 4.6% forgot to take methadone at the last weekend prior to the survey. Approximately 37.3% patients reported forgetting medication within the last 3 months. The mean percentage of adherence was 91.9% (SD = 10.5%). With the threshold 90% for optimal adherence, 52.8% reported optimal adherence in the last 7 days prior to the survey. Meanwhile, 34.4% were classified optimal adherence according to the standard of MoH.

**Table 4:** Pattern of MMT adherence among MMT patients

Characteristics	MMT duration ≤12 months		MMT duration >12 months		Total		*p*-value
	*n*	%	*n*	%	*n*	%	
Number of days missing doses in the last 4 days^a^							
• None	91	89.2	128	92.1	219	90.9	0.18^¶^
• 1 day	9	8.8	6	4.3	15	6.2	
• 2–4 days	2	2.0	5	3.6	6	2.9	
Forget to take doses at the last weekend^b^							
• Yes	5	4.9	6	4.3	11	4.6	0.69^†^
• No	97	95.1	131	94.2	228	94.6	
• Do not remember	0	0.0	2	1.4	2	0.8	
Last time that missing doses in the last 3 months^c^							
• 1 week ago	9	8.8	5	3.6	14	5.8	<0.01^¶^
• 2 weeks ago	4	3.9	5	3.6	9	3.7	
• 3–4 weeks ago	6	5.9	8	5.8	14	5.8	
• 1–2 months ago	5	4.9	9	6.5	14	5.8	
• 2–3 months ago	5	4.9	34	24.5	39	16.2	
• None	70	68.6	67	48.2	137	56.9	
• Do not remember	3	2.9	11	7.9	14	5.8	
Adherence (MoH’s standard)^d^							
• Sub-optimal adherence	58	56.9	100	71.9	158	65.6	0.02^¶^
• Optimal adherence	44	43.1	39	28.1	83	34.4	
Adherence (VAS)^e^							
• Sub-optimal adherence (<90%)	42	43.3	66	50.0	108	47.2	0.32^¶^
• Optimal adherence (≥90%)	55	56.7	66	50.0	121	52.8	
	Mean	SD	Mean	SD	Mean	SD	*p*-value
Self-assessed adherence (VAS)^f^	92.9	9.5	91.1	11.1	91.9	10.5	0.25^††^

^*¶*^ *χ* ^2^ *;* ^†^ *fisher’s exact test;* ^††^ *T-test*

^a^Pearson chi-squared test = 2.5157; ^b^Fisher’s exact test = 0.688; ^c^Pearson chi-squared test = 23.7634;

^d^Pearson chi-squared test = 5.9249; ^e^Pearson chi-squared test = 1.0075; ^f^T-test = 1.2880 (df = 227)

Regression models in Table 5 show that higher age, higher EQ-VAS, reminded by family members were positively associated with higher VAS score of adherence, while higher duration of MMT was associated with lower adherence score. People who were farmers and belonged to the middle-income group were less likely to have optimal adherence. Otherwise, having problems in usual activities and reminded by family members were positively associated with optimal adherence.

**Table 5:** Factor associated with non-adherence among respondents

Characteristics	Missing dose in the last 4 days		Missing dose at last weekend		Adherence (VAS)		Optimal adherence (MoH standard)	
	OR	95% CI	OR	95% CI	Coef.	95% CI	OR	95% CI
Age					0.44**	0.08; 0.79	1.03	0.99; 1.09
Education (vs < High school)								
• High school	3.81**	1.04; 13.94						
• >High school					10.79	−3.26; 24.85		
Employment (vs. Unemployment)								
• Self-employed	0.28**	0.08; 0.97						
• Worker/Farmer							0.11**	0.02; 0.67
• Other							0.24*	0.05; 1.24
HIV status (vs. Negative)								
• Positive	0.20*	0.04; 1.09	0.04	0.00; 2.47	8.26	−4.35; 20.86		
Income quintile (vs. poorest)								
• Poor	5.56**	1.09; 28.35	2.70	0.65; 11.25			0.40*	0.15; 1.04
• Middle							0.33**	0.13; 0.88
• Rich	2.99	0.61; 14.76						
• Richest	4.14*	0.82; 20.91					0.51	0.19; 1.38
Location (Son Duong vs. Tuyen Quang)							0.51	0.21; 1.22
Number of drug rehabilitation (vs. None)								
• 1 time	0.10***	0.02; 0.52					3.97**	1.23; 12.83
• 2 times							3.03*	0.86; 10.61
• > 2 times	0.33*	0.10; 1.15	0.14*	0.01; 1.38			2.84*	0.89; 9.12
Having problem in self-care (Yes vs No)	0.03**	0.00; 0.94	2.12	0.25; 18.28				
Having problem in usual activities (Yes vs. No)					7.41	−0.02; 14.84	3.74**	1.16; 12.07
Pain/Discomfort (Yes vs No)	0.11*	0.01; 1.53	6.48**	1.43; 29.42			0.26	0.05; 1.41
Anxiety/Depression							0.27	0.05; 1.35
EQ index	0.01**	0.00; 0.60					0.02**	0.00; 0.88
EQ VAS					0.22**	0.03; 0.42		
MMT duration (months)	1.00	0.94; 1.07	0.95	0.87; 1.04	−0.32**	−0.62; −0.01	0.97	0.93; 1.01
Adherence supporting measures								
• Mobile phone (Yes vs No)	0.09**	0.01; 0.96					0.37*	0.14; 1.02
• Reminded by family member (Yes vs. No)					13.02***	6.65; 19.39	2.75**	1.24; 6.08
ARV treatment (Yes vs. No)			0.08	0.00; 3.06	9.31	−3.55; 22.16		
Current smoking (Yes vs No)	0.38	0.12; 1.27	0.16**	0.03; 0.79				
Current drug use (Yes vs. No)					−5.77	−13.13; 1.58		

*** p < 0.01, ** p < 0.05, * p < 0.1

We also found that higher education and higher economic condition were related to higher likelihood of missing doses in the last 4 days. Meanwhile, being self-employed, having problems in self-care, higher EQ index, higher number of drug rehabilitation and support adherence by using mobile phone were inversely associated with missing doses in the last 4 days. Suffering pain/discomfort increased the risk of missing a dose at last weekend while being current smoker decreased this risk.

## Discussion

To our knowledge, this is the first study exploring the adherence patterns in MMT patients in a Vietnamese mountainous setting. This study indicates a high rate of sub-optimal adherence to MMT among respondents, which were influenced by various determinants regarding socio-economic, health, and social aspects. The study also reveals the positive effects of family and mobile phone supports in ensuring medication adherence. Findings of this study could be used to suggest potential solutions to promote adherence among MMT patients in Vietnam.

In this study, we found that the rate of suboptimal adherence according to MoH standard among MMT patients was 65.6%. This finding was much higher than the previous research in two other epicenters of Vietnam namely Hai Phong and Ho Chi Minh city, which revealed that after 24 months, the missing dose rate for 1–2 days was 41.4% [15]. This difference can be explained by the fact that Tuyen Quang is a mountainous province with the predominance of mountains, hills, and valleys. This geographical barrier reduced the accessibility of MMT service, resulting in the poor adherence among patients. Our result was also consistent with previous studies in Asia and Europe, which indicate high levels of poor adherence among MMT patients [8, 12-14]. Noticeably, we observed that the degree of adherence decreased disproportionately with the duration of MMT, which was similar to the previous studies in Vietnam [15] and France [8]. This phenomenon could be explained that when people had a long-term duration of treatment, they believed in their capacity for completely quitting drug use; thus, they were more likely to withdraw the program [25]. However, poor adherence can substantially increase the risk of drug relapse and predict the treatment failure [15]. Therefore, this result implies a need for monitoring patients’ adherence and providing counseling to the patients timely in order to assure the success of treatment.

Socio-economic characteristics of MMT patients were found to be associated with the treatment adherence. For example, people with older age, low education, and low income were less likely to report treatment non-adherence. Younger respondents, as in previous studies, might be more easily influenced by their peers’ drug use [13, 26]. Moreover, people who were workers or with high education level and high income might confront the conflict between their working time and the time spent on treatment; long distance between their workplace and MMT clinic; and drug-related stigma among their colleagues [13, 27, 28]. Therefore, these features should be considered by physicians when initiating methadone treatment in order to offer appropriate management and counseling service to such vulnerable populations for improving medication adherence.

Regarding health status, a previous study found that patients with depression were more likely to not adhere to treatment [8]. Our results contributed the understanding of strong relationships between HRQOL and treatment adherence. Specifically, pain/discomfort and anxiety/depression were inversely related to the adherence, while the EQ-5D index and EQ-VAS were positively associated with the adherence. Since having psychological and physical problems were known to increase the risk of drug relapse and treatment failure [29, 30], these findings emphasized the importance of identifying needs of patients aiming to provide comprehensive clinical care and counseling during the course of MMT. In addition, the current results show a potential of using EQ-5D-5 L to predict the medication adherence, that can be applied and validated in further studies.

In this study, we also found that patients who were current smokers were less likely to miss the dose in the last 4 days (*p* > 0.05) or miss the dose at last weekend (*p* < 0.05). Previous literature proved that methadone could interact with nicotine to generate the pleasing effect for MMT patients [31], which could help patients to ease the cravings of opiate (heroin or cocaine) consumptions [31]. Moreover, this interaction could escalate euphoria and reduce mental issues namely restlessness, irritability, and depression [32, 33]. As a result, smoking could motivate the methadone adherence among patients [34, 35]. However, in contrast to these short-term benefits, the long-term harms of this interaction are placing patients at high risk of morbidities, mortality and poor health status among patients who smoked compared to their counterparts [36-38]. Therefore, it is important to provide counseling to smoker patients which motivate them quitting smoke but keeping treatment adherence.

Notably, we found critical results that could serve as effective approaches to promote adherence among MMT patients. First, although most of the respondents preferred self-help as the main adherence support, patients who used mobile phone support had a higher likelihood of adhering MMT. However, we observed that only 18.3% patients used mobile phone alarm to support adherence. With the popularity and indispensable role of mobile phones in the modern life, the automated alarm of mobile phones could help to remind patients irrespective of settings. Particularly, mobile phones are an acceptable and effective tool for self-employed patients due to their high mobility and low instability, who required a tool that they could bring along and access in anywhere. Mobile phones have been shown to be effective in reminding ART adherence among HIV positive patients [39, 40]. Along with mobile phones, the positive association between family support and medication adherence has an important implication. Zhou et al. found that people not living with family were more likely to drop out the treatment [13]. Therefore, the involvement of family members, particularly wife/husband/lover of patients, played an integral part in supporting patients to overcome the clinical, economic, and social barriers, as well as remind daily treatment to ensure treatment adherence [40].

This study has several limitations that should be acknowledged. First, MMT adherence was self-reported data, which might be susceptible to recall bias and social desirability response bias [41]. Second, the convenience sampling method limited the generalizability of our findings to the MMT patients in other mountainous settings. Moreover, causal relations between MMT adherence and its determinants could not be drawn due to the use of cross-sectional design, which suggests further experiments to identify clearly these relationships. Finally, some clinical and services-related factors such as methadone dose, quality of services and patients’ satisfaction were not included in this study, which required further studies to fulfill the gap of knowledge in order to enhance the quality and effectiveness of MMT program in Vietnam.

## Conclusion

In conclusion, this study found a high rate of sub-optimal adherence among MMT patients in a mountainous setting in Vietnam. Measuring optimal adherence by several simple items could be used periodically to monitor the treatment adherence in the clinical setting, especially in mountainous areas. Family and mobile phone support would have a potential role in supporting patients to adhere to treatment.

## Abbreviation

AIDS: Acquired Immune Deficiency Syndrome
ART: Antiretroviral therapy
AUDIT-C: Alcohol Use Disorders Identification Test – Consumption
EQ-5D-5 L: EuroQol – 5 dimensions – 5 levels
HIV: Human Immunodeficiency Virus
HRQOL: Health-related quality of life
MMT: Methadone maintenance treatment
PWID: People who inject drugs
VAS: Visual analogue scale
